# Supplementary material for: Socially meaningful visual context either enhances or inhibits vocalisation processing in the macaque brain
Source: Nat Commun. 2022 Aug 19;13:4886. doi: 10.1038/s41467-022-32512-9 (PMC9391382; doi:10.1038/s41467-022-32512-9)
Supplement: Supplementary file 1 — Supplementary Information [file 41467_2022_32512_MOESM1_ESM.pdf]

Supplementary Material (tables and figures)

Supplementary Table 1: **Percentage of signal change (PSC) across all atlas ROIs of both hemispheres (median + se), comparing visual, auditory congruent and incongruent conditions vs fixation for the face context (F+ and F- combined).** This is a companion table to Figure 2 and Supplementary Figure S3. ROIs are selected based on whether they contain significantly activated voxels as per the analysis presented in Figures 2, 3 and/or 4 and are extracted from the CIVM atlas. They are outlined in Supplementary Figure 3. In bold, significant activations relative to fixation baseline, p<0.001 (Two-sided Wilcoxon non-parametric test, p<0.05, adjusted for multiple comparisons). Selected ROIs are: V1, V2, V3d, V3v, V3A, area 2, area 3b, Depth IntraParietal area (DIP), Pea, POa external part, POa internal part, V4d, V4v, V4t, Parietal area PG, Area PG associated region of the STS (PGa), lateral auditory koniocortex (AKL), medial auditory koniocortex (AKM), fundus of the Superior Temporal Sulcus (f STS), Medial Superior Temporal area (MST), Medial Temporal area (v5) (MT),Retroinsular area (ReI), Paraauditory area caudal part (PaAC), Parainsular cortex lateral part (PaIL), Prokoniocortex medial part (ProKM), Temporal area TE occipital part (TEO), Temporal area TE occipital medial part (TEOm), TEa, Intraparietal sulcus associated area in the STS (IPa), Temporal parietooccipital associated area in STS (TPO), TPO caudal part (TPOC), area 45A, area 45B, area 9/46, area 47 (old_12) orbital part).

	Hemisphere	Face context					
		Vi vs Fx		AC vs Fx		AI vs Fx	
		PSC	se	PSC	se	PSC	se
V1	R	0,82	0,25	0,56	0,21	0,49	0,31
	L	**1,14**	0,23	0,93	0,22	0,15	0,32
V2	R	**0,92**	0,17	**0,95**	0,14	0,15	0,18
	L	**0,89**	0,14	0,77	0,12	-0,03	0,15
V3d	R	**0,56**	0,09	0,62	0,10	0,06	0,10
	L	**0,95**	0,13	0,68	0,12	0,08	0,12
V3v	R	0,50	0,30	0,54	0,27	-0,05	0,35
	L	0,46	0,20	0,57	0,17	0,09	0,22
V3A	R	**0,71**	0,13	0,50	0,10	0,10	0,13
	L	**0,80**	0,13	0,47	0,12	-0,23	0,13
area 2	R	0,14	0,30	-0,02	0,32	-0,16	0,35
	L	0,20	0,27	0,01	0,30	-0,30	0,33
area 3b	R	0,25	0,11	0,28	0,13	-0,01	0,12
	L	0,19	0,11	0,26	0,12	0,02	0,11
Depth IntraParietal area (DIP)	R	0,30	0,07	0,20	0,07	0,10	0,08
	L	0,14	0,09	0,22	0,08	0,00	0,09
PEa	R	0,23	0,13	0,20	0,10	-0,07	0,12
	L	0,20	0,15	0,32	0,13	-0,12	0,15
POa external part	R	-0,10	0,21	-0,09	0,21	0,10	0,23
	L	0,03	0,22	0,34	0,25	-0,12	0,26
POa internal part	R	0,22	0,09	0,41	0,08	0,09	0,09
	L	0,22	0,10	0,39	0,08	0,01	0,09
V4d	R	**1,15**	0,28	**0,71**	0,24	0,09	0,29
	L	**1,08**	0,26	0,52	0,24	-0,13	0,28
V4v	R	0,73	0,32	0,11	0,32	-0,08	0,38
	L	0,51	0,25	0,38	0,23	0,06	0,29
V4t	R	**1,15**	0,28	0,71	0,24	0,09	0,29
	L	**1,08**	0,26	0,52	0,24	-0,13	0,28
Parietal area PG	R	0,09	0,75	-0,20	0,80	-0,40	0,91
	L	0,07	0,72	0,20	0,82	0,11	0,92
Area PG associated region of the STS (PGa)	R	**0,72**	0,21	0,27	0,21	-0,09	0,21
	L	**1,00**	0,18	**0,55**	0,18	0,18	0,18
lateral auditory koniocortex (AKL)	R	0,29	0,23	**0,81**	0,27	0,42	0,27
	L	0,46	0,33	**0,68**	0,36	0,33	0,37
medial auditory koniocortex (AKM)	R	0,27	0,17	**0,51**	0,19	0,30	0,19
	L	0,58	0,35	0,70	0,40	0,13	0,41
fundus of the Superior Temporal Sulcus (f STS)	R	**1,02**	0,16	**0,68**	0,17	0,30	0,19
	L	**1,04**	0,22	**0,83**	0,26	0,12	0,28
Medial Superior Temporal area (MST)	R	0,43	0,16	0,24	0,15	-0,04	0,19
	L	0,38	0,12	0,33	0,11	-0,06	0,13
Medial Temporal area (v5) (MT)	R	**1,09**	0,18	**0,66**	0,18	0,12	0,18
	L	**0,75**	0,12	**0,59**	0,15	-0,06	0,13
Retroinsular area (ReI)	R	0,33	0,21	0,21	0,22	-0,15	0,21
	L	0,44	0,13	0,32	0,11	0,18	0,11
Paraauditory area caudal part (PaAC)	R	0,21	0,09	**0,66**	0,11	0,03	0,10
	L	0,40	0,21	**0,66**	0,22	0,27	0,22
Parainsular cortex lateral part (PaIL)	R	-0,07	0,25	0,46	0,27	0,28	0,28
	L	0,27	0,19	0,50	0,22	0,29	0,18
Prokoniocortex medial part (ProKM)	R	0,47	0,36	0,37	0,39	0,25	0,45
	L	0,57	0,31	0,46	0,37	0,23	0,38
Temporal area TE occipital part (TEO)	R	**1,09**	0,39	0,64	0,40	0,02	0,46
	L	**1,07**	0,38	**0,62**	0,38	0,06	0,40
Temporal area TE occipital medial part (TEOm)	R	**0,85**	0,39	0,36	0,41	0,15	0,47
	L	**0,94**	0,30	0,33	0,58	0,00	0,65
TEa	R	**1,10**	0,15	0,52	0,15	0,11	0,14
	L	**1,07**	0,14	**0,71**	0,15	0,14	0,12
Intraparietal sulcus associated area in the STS (IPa)	R	**0,77**	0,24	0,19	0,24	0,00	0,24
	L	**1,16**	0,17	**0,64**	0,15	0,29	0,15
Temporal parietooccipital associated area in STS (TPO)	R	**0,78**	0,21	**0,80**	0,20	0,41	0,21
	L	**0,86**	0,23	0,68	0,25	0,12	0,24
TPO caudal part (TPOC)	R	**1,01**	0,22	**0,71**	0,22	0,11	0,23
	L	**0,78**	0,17	0,69	0,21	0,03	0,17
area 45A	R	**0,77**	0,12	**0,70**	0,13	0,00	0,12
	L	**0,86**	0,10	**0,47**	0,11	0,21	0,09
area 45B	R	**0,72**	0,14	**0,60**	0,11	0,20	0,14
	L	**0,68**	0,14	0,31	0,12	-0,11	0,13
area 9/46	R	**0,70**	0,11	**0,58**	0,12	0,27	0,13
	L	**1,26**	0,29	0,53	0,28	0,29	0,31
area 47 (old_12) orbital part	R	0,27	0,10	0,28	0,10	0,09	0,10
	L	0,22	0,25	0,10	0,28	-0,07	0,31

18

Supplementary Table 2: **Percentage of signal change (PSC) across all selected atlas ROIs of both hemispheres (median + se), comparing visual, auditory congruent and incongruent conditions vs fixation for F+ and F- contexts separately.** This is a companion table to Figure 3 and Supplementary Figure 7. all as in Supplementary Table 1.

	Hemisphere	F+						F-					
		Vi vs Fx		AC vs Fx		AI vs Fx		Vi vs Fx		AC vs Fx		AI vs Fx	
		PSC	se	PSC	se	PSC	se	PSC	se	PSC	se	PSC	se
V1	R	0,62	0,31	0,56	0,30	0,13	0,44	1,00	0,38	0,53	0,30	0,75	0,44
	L	**1,10**	0,30	**0,83**	0,29	0,15	0,44	**1,33**	0,37	0,73	0,31	0,18	0,47
V2	R	**0,90**	0,20	0,87	0,22	0,20	0,20	**1,05**	0,26	**0,98**	0,18	0,15	0,25
	L	**0,84**	0,16	**0,77**	0,16	0,14	0,27	**1,14**	0,24	0,69	0,18	-0,10	0,23
V3d	R	**0,59**	0,13	**0,54**	0,14	0,26	0,15	**0,68**	0,13	**0,54**	0,13	0,04	0,13
	L	0,91	0,24	0,92	0,22	-0,12	0,22	**1,00**	0,14	0,51	0,12	0,10	0,13
V3v	R	0,50	0,41	1,09	0,37	-0,34	0,48	0,61	0,43	-0,07	0,38	0,33	0,50
	L	0,56	0,22	0,34	0,24	0,22	-0,28	0,59	0,32	0,53	0,26	0,29	0,34
V3A	R	0,68	0,18	0,72	0,12	0,15	0,13	**0,83**	0,21	0,33	0,14	-0,11	0,22
	L	**0,86**	0,21	**0,77**	0,21	-0,21	0,19	**0,74**	0,17	0,34	0,12	-0,24	0,17
area 2	R	-0,04	0,41	0,29	0,42	-0,28	0,45	0,42	0,44	-0,24	0,48	0,15	0,55
	L	-0,09	0,32	0,09	0,31	-0,52	0,32	0,58	0,45	-0,08	0,54	-0,05	0,63
area 3b	R	-0,12	0,12	0,30	0,14	0,00	0,12	0,47	0,19	0,16	0,22	-0,01	0,23
	L	0,08	0,15	0,14	0,19	-0,08	0,15	0,66	0,15	0,42	0,15	0,22	0,14
Depth IntraParietal area (DIP)	R	0,24	0,12	0,27	0,11	0,13	0,12	0,30	0,09	0,20	0,09	0,09	0,11
	L	0,00	0,16	0,26	0,13	-0,21	0,14	0,24	0,10	0,16	0,11	0,16	0,12
PEa	R	-0,02	0,16	0,15	0,13	-0,07	0,13	0,34	0,20	0,22	0,14	-0,03	0,21
	L	-0,16	0,27	0,37	0,27	-0,37	0,27	**0,39**	0,14	**0,32**	0,11	-0,07	0,15
POa external part	R	-0,15	0,26	0,79	0,28	-0,06	0,29	0,68	0,33	-0,25	0,30	0,22	0,34
	L	-0,12	0,29	0,77	0,34	-0,36	0,33	0,61	0,33	-0,15	0,33	-0,01	0,38
POa internal part	R	0,16	0,15	0,41	0,13	0,08	0,14	0,33	0,12	**0,36**	0,09	0,09	0,13
	L	0,08	0,16	0,37	0,14	-0,16	0,14	**0,30**	0,11	**0,40**	0,09	0,09	0,12
V4d	R	**1,07**	0,28	0,80	0,36	-0,05	0,32	**1,27**	0,44	0,14	0,36	0,11	0,51
	L	**0,95**	0,24	**0,95**	0,25	-0,01	0,22	**1,24**	0,51	0,40	0,40	-0,17	0,58
V4v	R	0,64	0,43	0,78	0,38	-0,38	0,49	0,74	0,47	-0,45	0,51	0,45	0,59
	L	0,36	0,35	0,48	0,30	-0,32	0,36	**0,67**	0,35	0,05	0,36	0,40	0,44
V4t	R	0,80	0,36	**1,07**	0,28	-0,05	0,32	**1,27**	0,44	0,14	0,36	0,11	0,51
	L	**0,95**	0,24	**0,95**	0,25	-0,01	0,22	1,24	0,51	0,40	0,40	-0,17	0,58
Parietal area PG	R	-0,46	1,04	0,54	1,08	-0,59	1,15	0,63	1,07	-0,49	1,22	-0,23	1,44
	L	-0,02	0,88	0,67	0,94	0,11	0,96	0,33	1,18	-0,59	1,41	0,21	1,65
Area PG associated region of the STS (PGa)	R	0,74	0,24	**0,96**	0,25	-0,11	0,18	0,80	0,35	0,07	0,33	0,33	0,42
	L	**1,16**	0,24	**1,05**	0,23	0,12	0,19	0,88	0,27	0,17	0,25	0,27	0,34
lateral auditory koniocortex (AKL)	R	0,13	0,30	**0,89**	0,32	0,37	0,29	0,35	0,34	0,71	0,44	0,45	0,46
	L	0,07	0,53	**0,86**	0,36	0,09	0,54	0,39	0,48	**0,62**	0,38	0,38	0,49
medial auditory koniocortex (AKM)	R	0,18	0,30	**0,98**	0,32	0,18	0,29	0,37	0,17	0,26	0,21	0,36	0,24
	L	0,37	0,51	**0,94**	0,37	0,10	0,55	0,60	0,47	0,30	0,58	0,34	0,62
fundus of the Superior Temporal Sulcus (f STS)	R	**1,14**	0,26	**1,38**	0,25	0,30	0,22	**0,87**	0,19	0,42	0,21	0,32	0,32
	L	**1,07**	0,26	**1,48**	0,28	0,19	0,28	**1,04**	0,37	-0,03	0,39	-0,02	0,52
Medial Superior Temporal area (MST)	R	0,48	0,14	0,59	0,16	-0,21	0,20	0,34	0,31	-0,17	0,24	-0,01	0,32
	L	0,36	0,14	0,26	0,15	-0,24	0,15	0,41	0,20	0,33	0,16	0,07	0,22
Medial Temporal area (v5) (MT)	R	**1,21**	0,24	**1,01**	0,24	0,12	0,21	**1,09**	0,26	0,17	0,23	0,20	0,31
	L	**1,07**	0,22	**0,74**	0,19	0,16	0,15	**0,85**	0,19	**0,41**	0,18	0,02	0,26
Retroinsular area (ReI)	R	0,23	0,23	0,67	0,24	0,07	0,23	0,06	0,38	0,40	0,37	-0,24	0,36
	L	0,33	0,13	0,38	0,12	0,27	0,11	0,28	0,20	**0,62**	0,25	-0,01	0,22
Paraauditory area caudal part (PaAC)	R	0,10	0,13	**0,70**	0,14	0,09	0,13	0,26	0,13	**0,58**	0,17	-0,18	0,15
	L	0,38	0,30	**0,88**	0,34	0,26	0,32	0,51	0,29	0,53	0,28	0,26	0,30
Parainsular cortex lateral part (PaIL)	R	-0,11	0,13	0,49	0,16	0,26	0,15	-0,05	0,59	0,72	0,60	0,29	0,66
	L	0,31	0,36	**1,00**	0,39	0,35	0,36	0,17	0,19	0,16	0,23	0,29	0,15
Prokoniocortex medial part (ProKM)	R	0,39	0,40	0,81	0,42	0,36	0,45	0,54	0,61	-0,41	0,66	-0,06	0,83
	L	0,15	0,28	**0,77**	0,24	0,18	0,29	0,80	0,61	-0,32	0,67	0,23	0,79
Temporal area TE occipital part (TEO)	R	**1,07**	0,41	**1,15**	0,34	0,08	0,42	**1,09**	0,50	0,02	0,75	-0,01	0,89
	L	**1,07**	0,53	**1,34**	0,54	0,00	0,53	0,95	0,53	0,10	0,50	0,12	0,61
Temporal area TE occipital medial part (TEOm)	R	0,68	0,50	0,42	0,50	0,09	0,48	0,87	0,60	-0,13	0,68	0,35	0,85
	L	0,71	0,64	1,00	0,70	-0,22	0,66	1,22	0,79	-0,28	0,94	0,24	1,16
TEa	R	**1,06**	0,24	**1,20**	0,24	0,02	0,17	**1,10**	0,18	0,39	0,16	0,21	0,24
	L	**0,94**	0,24	**0,84**	0,22	-0,09	0,19	**1,10**	0,17	**0,69**	0,21	0,41	0,15
Intraparietal sulcus associated area in the STS (IPa)	R	**0,96**	0,29	**0,83**	0,29	-0,09	0,21	0,69	0,41	-0,16	0,35	0,28	0,49
	L	**1,24**	0,23	**1,16**	0,21	0,01	0,19	**1,09**	0,24	**0,42**	0,19	0,39	0,25
Temporal parietooccipital associated area in STS (TPO)	R	0,85	0,26	**1,23**	0,23	0,25	0,22	0,75	0,33	0,44	0,32	0,45	0,38
	L	0,83	0,34	**1,36**	0,34	-0,12	0,32	0,86	0,30	0,19	0,32	0,16	0,38
TPO caudal part (TPOC)	R	0,72	0,31	0,93	0,30	-0,36	0,28	**1,01**	0,30	0,39	0,31	0,41	0,37
	L	0,79	0,24	0,75	0,33	-0,02	0,19	0,78	0,24	0,49	0,25	0,36	0,30
area 45A	R	**0,78**	0,13	**0,87**	0,15	0,00	0,12	0,67	0,21	0,44	0,19	0,15	0,22
	L	**0,78**	0,16	0,60	0,17	-0,08	0,14	**0,87**	0,13	**0,41**	0,14	**0,42**	0,10
area 45B	R	**0,67**	0,19	**0,73**	0,18	0,19	0,19	**0,76**	0,21	**0,41**	0,13	0,19	0,20
	L	**0,65**	0,22	0,48	0,21	0,04	0,21	**0,72**	0,18	0,29	0,12	-0,11	0,15
area 9/46	R	0,62	0,18	0,78	0,16	0,13	0,16	**0,88**	0,14	0,37	0,18	0,37	0,20
	L	1,27	0,46	0,83	0,43	0,24	0,48	**1,26**	0,38	0,28	0,35	0,35	0,40
area 47 (old_12) orbital part	R	0,31	0,14	0,31	0,12	0,09	0,11	0,23	0,16	0,16	0,16	0,13	0,18
	L	0,19	0,33	0,29	0,38	-0,42	0,36	0,30	0,39	0,04	0,42	0,35	0,51

Supplementary Table 3: **Percentage of signal change (PSC) across all selected atlas ROIs of both hemispheres (median + se), comparing visual, auditory congruent and incongruent conditions vs fixation for social context (S1+, S1-, S2+, S2- combined).** This is a companion table to Figure 4 and Supplementary Figure 11. all as in Supplementary Table 1.

	Hemisphere	Social context					
		Vi vs Fx		AC vs Fx		AI vs Fx	
		PSC	se	PSC	se	PSC	se
V1	R	0,30	0,21	0,39	0,21	0,04	0,23
	L	0,43	0,19	0,35	0,19	0,32	0,19
V2	R	**0,57**	0,17	**0,55**	0,16	0,20	0,16
	L	**0,61**	0,10	**0,52**	0,10	0,09	0,10
V3d	R	**0,53**	0,15	**0,46**	0,17	0,02	0,13
	L	**0,68**	0,14	**0,36**	0,09	0,18	0,18
V3v	R	0,56	0,20	0,18	0,22	0,07	0,15
	L	**0,71**	0,28	0,23	0,18	0,00	0,20
V3A	R	**0,53**	0,07	**0,45**	0,08	0,20	0,07
	L	**0,68**	0,09	**0,37**	0,10	0,19	0,09
area 2	R	0,10	0,38	-0,14	0,32	0,07	0,26
	L	0,05	0,33	-0,11	0,26	0,05	0,23
area 3b	R	0,16	0,13	-0,01	0,13	-0,02	0,11
	L	0,11	0,17	-0,10	0,16	0,17	0,13
Depth IntraParietal area (DIP)	R	0,08	0,08	0,15	0,08	-0,01	0,08
	L	0,23	0,06	0,12	0,06	0,15	0,05
PEa	R	**0,46**	0,19	0,16	0,14	-0,06	0,12
	L	0,49	0,31	0,05	0,23	0,11	0,21
POa external part	R	0,18	0,30	0,04	0,22	0,01	0,19
	L	0,29	0,44	-0,12	0,37	0,09	0,32
POa internal part	R	0,36	0,06	0,37	0,07	0,08	0,07
	L	0,35	0,07	0,18	0,07	0,13	0,07
V4d	R	**0,73**	0,12	0,27	0,12	0,28	0,10
	L	**0,93**	0,11	**0,76**	0,11	0,10	0,09
V4v	R	0,64	0,46	-0,15	0,36	-0,08	0,31
	L	0,57	0,46	-0,03	0,36	-0,06	0,32
V4t	R	**0,73**	0,12	0,27	0,12	0,28	0,10
	L	**0,93**	0,11	**0,76**	0,11	0,10	0,09
Parietal area PG	R	-0,01	0,82	-0,15	0,65	-0,21	0,55
	L	0,21	0,88	-0,32	0,70	-0,14	0,60
Area PG associated region of the STS (PGa)	R	0,19	0,08	0,19	0,09	0,10	0,08
	L	**0,40**	0,06	0,23	0,06	0,08	0,06
lateral auditory koniocortex (AKL)	R	-0,06	0,15	0,22	0,12	0,08	0,15
	L	-0,08	0,16	0,08	0,16	0,28	0,12
medial auditory koniocortex (AKM)	R	-0,03	0,19	-0,03	0,17	0,07	0,14
	L	-0,13	0,17	0,14	0,18	0,17	0,14
fundus of the Superior Temporal Sulcus (f STS)	R	**0,51**	0,13	**0,38**	0,11	0,16	0,09
	L	**0,57**	0,16	0,29	0,16	0,14	0,13
Medial Superior Temporal area (MST)	R	0,27	0,17	0,00	0,13	0,01	0,11
	L	**0,53**	0,14	0,26	0,11	0,10	0,12
Medial Temporal area (v5) (MT)	R	0,43	0,14	0,33	0,10	0,07	0,10
	L	0,44	0,10	0,32	0,09	0,06	0,08
Retroinsular area (ReI)	R	0,24	0,09	0,08	0,10	-0,03	0,09
	L	0,29	0,10	0,16	0,09	0,19	0,07
Paraauditory area caudal part (PaAC)	R	0,03	0,11	**0,49**	0,10	0,05	0,09
	L	-0,20	0,21	0,23	0,20	0,20	0,15
Parainsular cortex lateral part (PaIL)	R	-0,21	0,18	0,12	0,18	0,03	0,15
	L	0,00	0,12	0,34	0,13	0,24	0,11
Prokoniocortex medial part (ProKM)	R	0,12	0,15	0,08	0,18	0,00	0,13
	L	0,07	0,11	0,12	0,13	0,10	0,09
Temporal area TE occipital part (TEO)	R	**0,93**	0,33	0,10	0,29	-0,11	0,25
	L	**1,05**	0,20	**0,61**	0,18	0,18	0,16
Temporal area TE occipital medial part (TEOm)	R	0,34	0,15	0,17	0,15	0,07	0,11
	L	0,38	0,20	0,30	0,16	0,07	0,14
TEa	R	**0,51**	0,12	0,29	0,12	0,11	0,11
	L	**0,59**	0,15	0,34	0,14	0,11	0,12
Intraparietal sulcus associated area in the STS (IPa)	R	0,35	0,12	0,22	0,12	0,03	0,12
	L	**0,56**	0,13	**0,42**	0,11	0,04	0,10
Temporal parietooccipital associated area in STS (TPO)	R	0,18	0,10	0,26	0,12	0,15	0,09
	L	**0,43**	0,13	0,33	0,12	0,16	0,10
TPO caudal part (TPOC)	R	**0,58**	0,20	0,17	0,17	-0,01	0,14
	L	**0,71**	0,20	0,36	0,18	0,12	0,16
area 45A	R	**0,52**	0,19	0,18	0,14	0,09	0,13
	L	**0,42**	0,19	0,27	0,15	0,21	0,14
area 45B	R	**0,57**	0,10	0,18	0,09	0,11	0,09
	L	**0,48**	0,13	0,21	0,11	0,08	0,10
area 9/46	R	**0,67**	0,32	0,48	0,26	-0,01	0,23
	L	**0,74**	0,42	0,12	0,35	0,16	0,31
area 47 (old_12) orbital part	R	0,05	0,08	-0,02	0,08	0,11	0,07
	L	0,25	0,20	-0,09	0,20	0,02	0,15

29

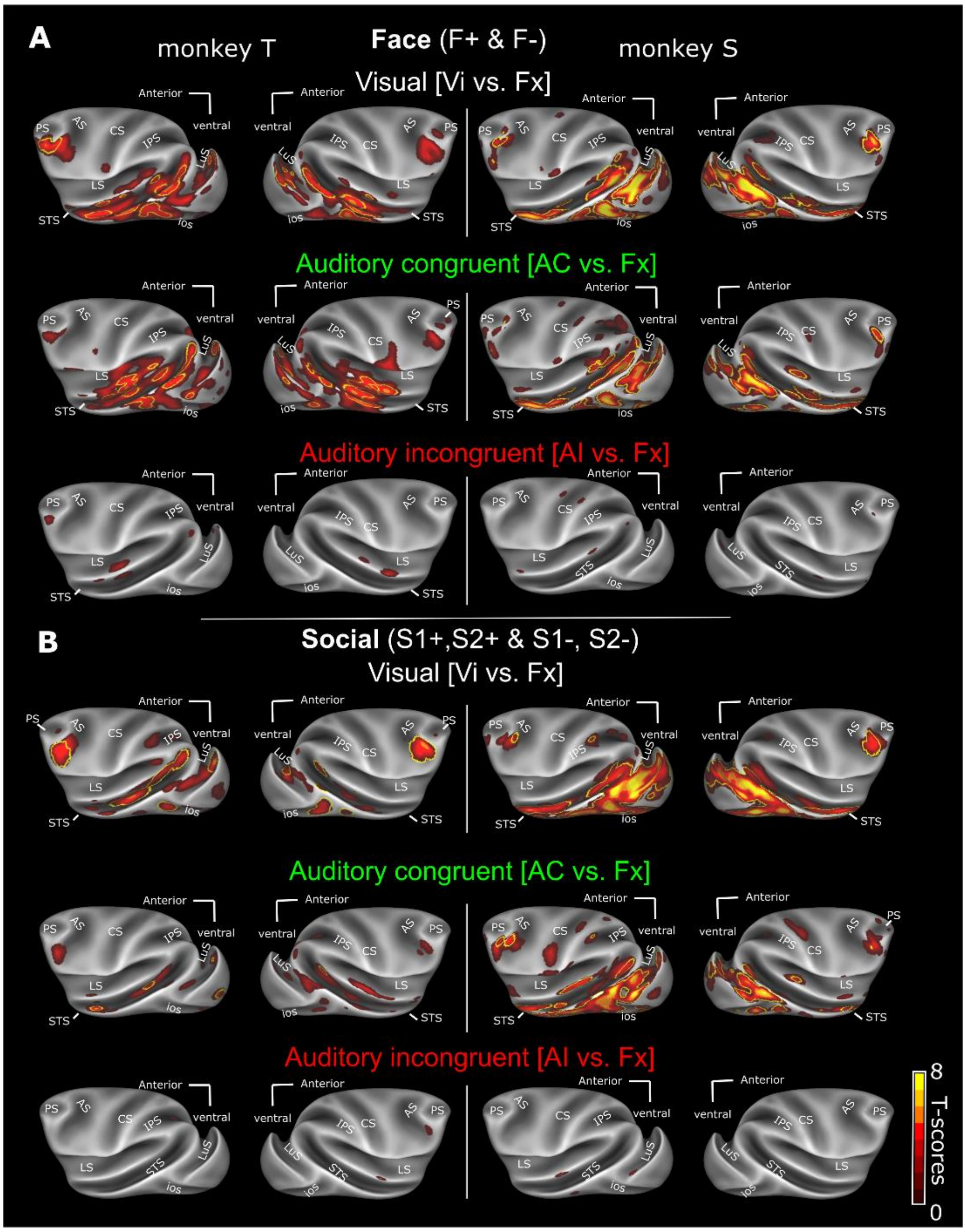

Supplementary Figure 1. Individual whole-brain activations for monkey T (left) and monkey S (right), for FACE contexts (F+ & F-, top) and SOCIAL contexts (S1+, S2+ & S1-, S2-, bottom) for the visual (white, Vi vs. Fx), auditory congruent (green, AC vs. Fx) and auditory incongruent (red, AI vs. Fx) contrasts. Darker shades of red indicate level of significance at p<0.001 uncorrected, t-score >3.09Lighter shades of yellow and brown outlines indicate level of significance at p<0.05 FEW corrected, t-score>4.6, DF= [1 2611] for Face, monkey S and T, DF= [1, 5107] for Social Monkey S and DF= [1, 5237] for Social Monkey T. ios: Inferior Occipital Sulcus; LS: Lateral Sulcus; STS: Superior Temporal Sulcus; CiS: Cingulate Sulcus; LuS: Lunate Sulcus; IPS: Intraparietal Sulcus; PS: Precentral Sulcus; CS: Central Sulcus; AS: Arcuate Sulcus; OrbS: Orbital Sulcus.

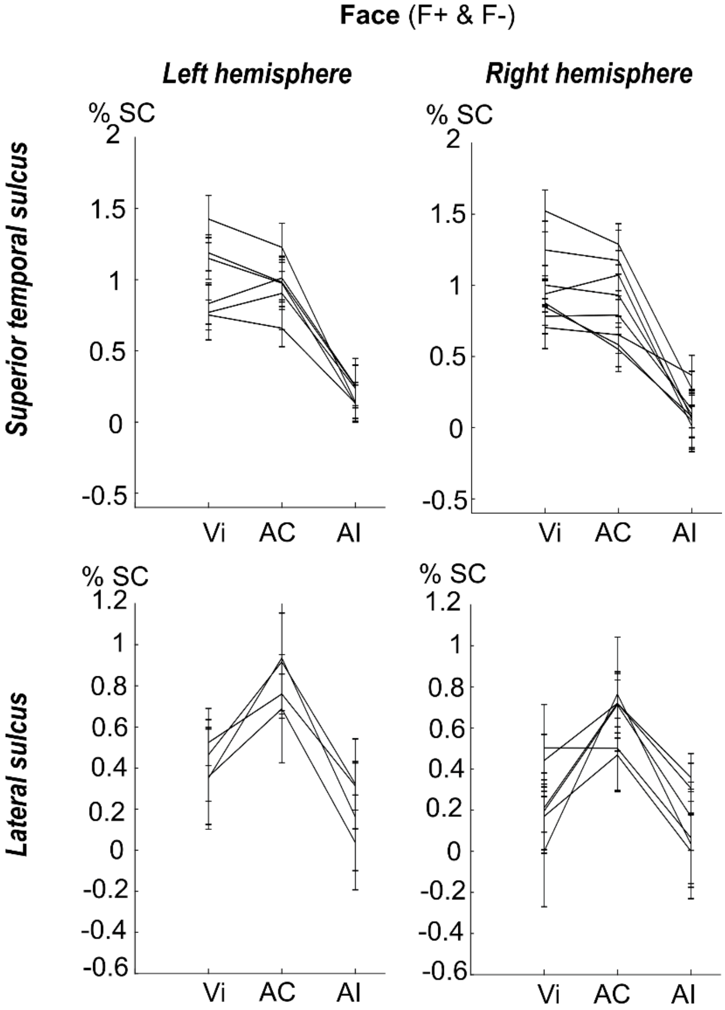

Supplementary Figure 2: Percentage of signal change (%SC) for selected left and right hemisphere ROIs in the lateral sulcus and in the superior temporal sulci. ROIs are 1.5mm spheres located at local peak activations of these two sulci. ROI location in the each of the left and right STS and LS is described in the bottom flat maps of figure 6. %SC (mean +/- se) are presented for each ROI (8 in right STS, 6 in left STS, 4 in left and 6 in right lateral sulcus) for the face context for the contrast Visual vs Fixation (Vi), Auditory congruent vs Fixation (AC) and Auditory incongruent vs Fixation (AI).

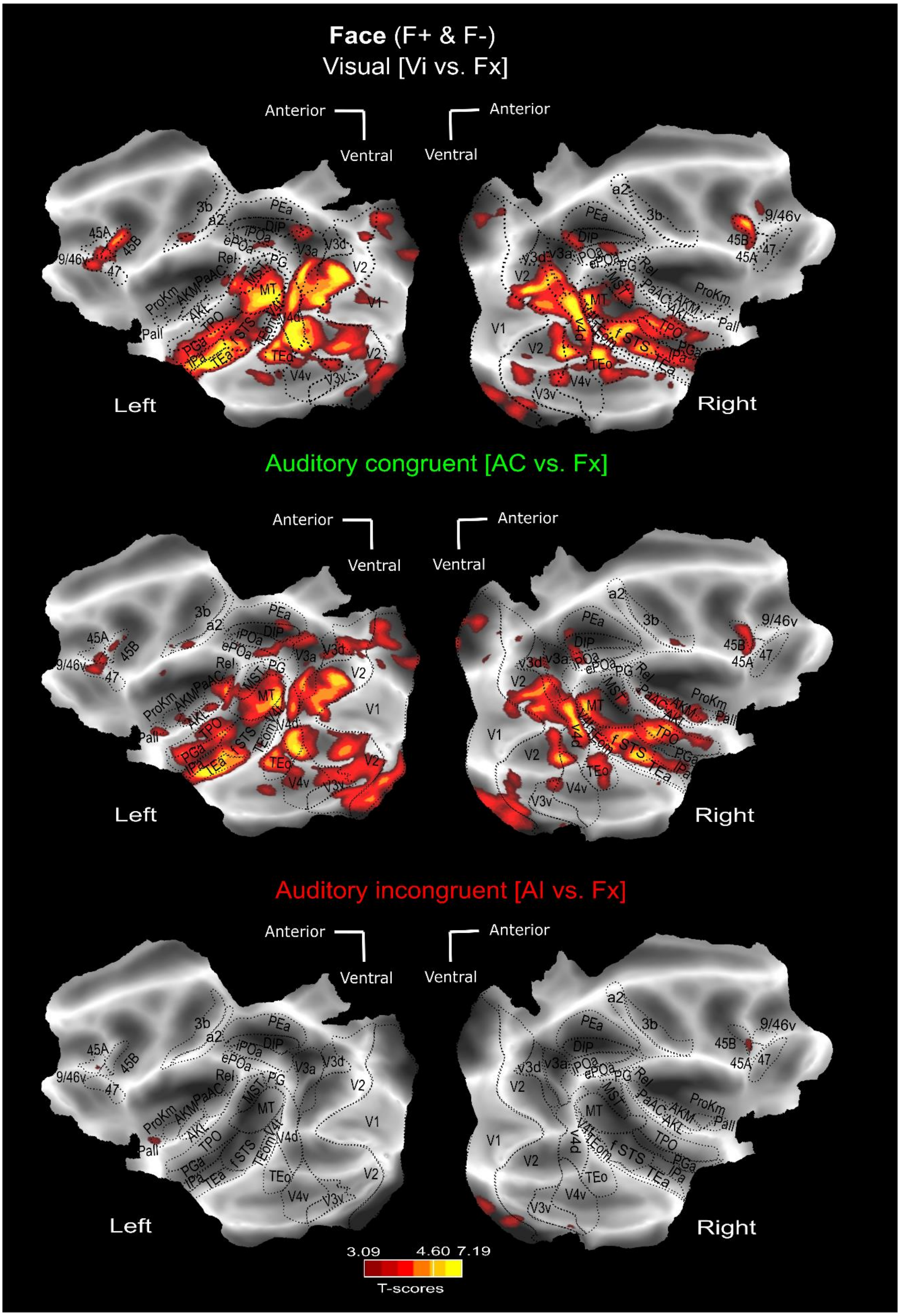

Supplementary Figure 3. All as in Figure 2, with the CIVM atlas (https://scalablebrainatlas.incf.org/macaque/CBCetal15, Calabrese et al., 2015) overlaid onto the cortical activations. PSC (median +/-s.e.) in each anatomical ROI described in Supplementary Table 1.

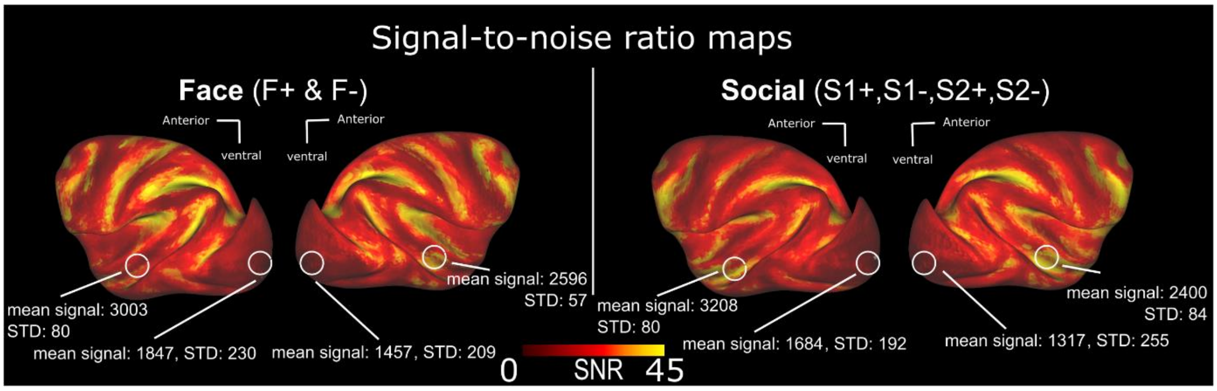

Supplementary Figure 4. Temporal signal to noise maps for the pooled face contexts (left) and the pooled social contexts (right). Top: inflated left and right hemispheres. Bottom: flat maps of left and right hemispheres. SNR is calculated by dividing the mean signal by its standard deviation. Low SNR in the occipital cortex is accounted for by both low signal and high signal variability (Face context: left: mean signal: 1847, STD: 230; right: mean signal: 1457, STD: 209; Social context: left: mean signal: 1684, STD: 192; right: mean signal: 1317, STD: 255). This is to be compared to areas of high SNR such as the STS, which is characterized by high signal mean and low signal variability (Face context: left: mean signal: 3003, STD: 80; right: mean signal: 2596, STD: 57; Social context: left: mean signal: 3208, STD: 80; right: mean signal: 2400, STD: 84. Please note that in spite of these lower SNR in the occipital cortex, %SC based on an atlas defined ROIs are occasionally significant for the Visual vs. fixation contrast, and (less so) for the Auditory congruent vs. fixation contrast, in V1, V2, V3 and V4 (see Supplementary Tables 1, 2 and 3).

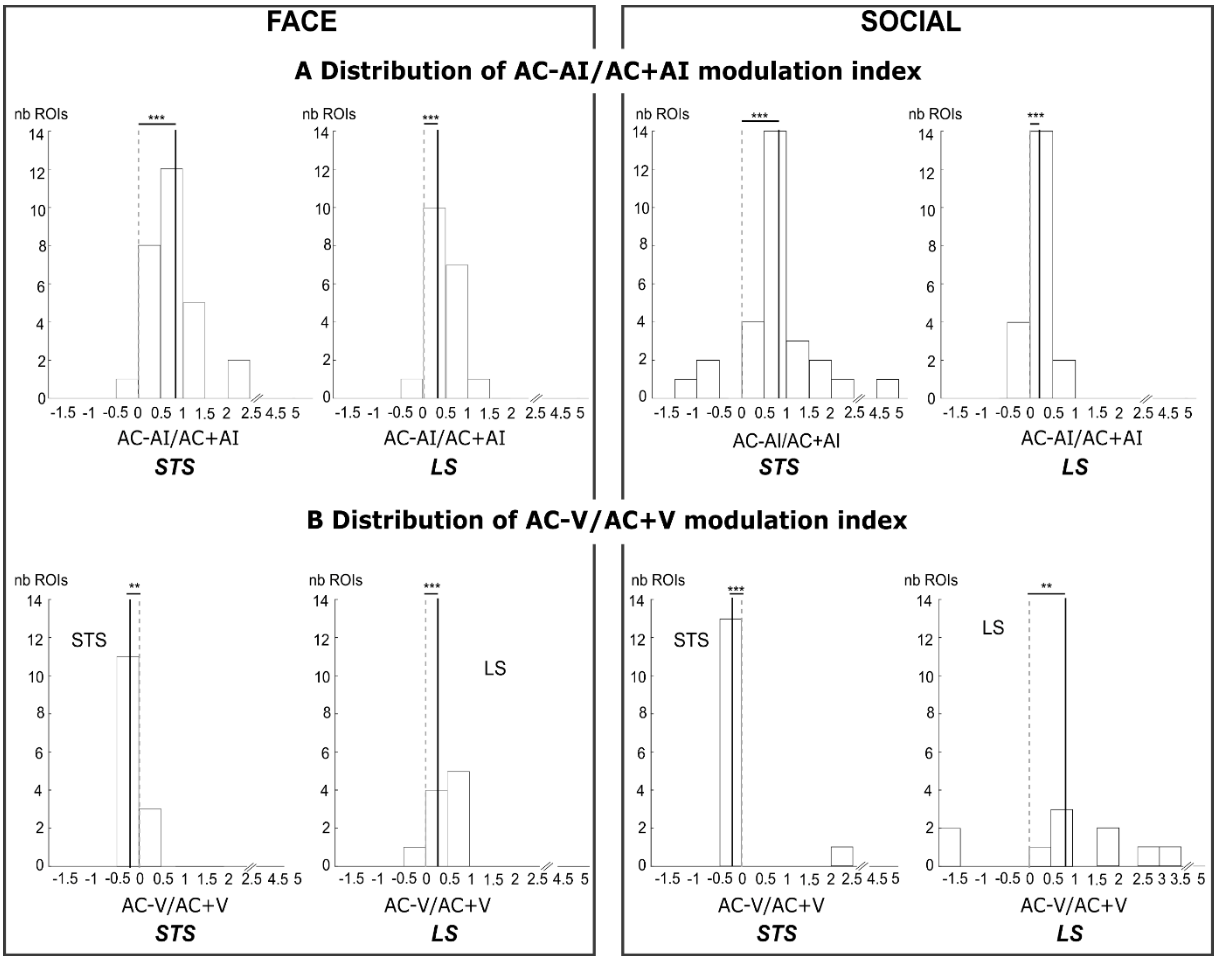

Supplementary Figure 5. Distribution of modulation index of percentage signal change (%SC) for the AC condition relative to baseline compared to the AI condition relative to baseline (A) and for the AC condition relative to baseline compared to the Visual condition relative to baseline (B), for each of the STS and LS, and each of the face and social runs, computed on individual ROIs across all runs. Thin line represents the zero index (no modulation of the hemodynamic response). The thick vertical line represents the median of the modulation index distribution. Statistical differences relative to zero are indicated as follows: ***, p<0.001; **, p<0.01; n.s., p>0.05 (Wilcoxon two-sided non-parametric test, A) AC/AI index: FACE: STS: n= 28, Z=4.78, p=1.92e-10<0.001 LS: n= 10, Z=3.99, p=3.9e-06<0.001; SOCIAL: STS: n=28, Z=4.79, p=7.1e-08<0.001 LS: n=10, Z=2.89, p=5.9e-04<0.001. B) AC/V index: FACE: STS: n= 28, Z=2.38, p=0.006<0.01, STSLS: n=10, Z=3.19, p=<0.0014; SOCIAL: STS: n= 28, Z=2.73, p 4.1e-05<0.001 LS: n=10, 4.10, p<0.001=0.01).

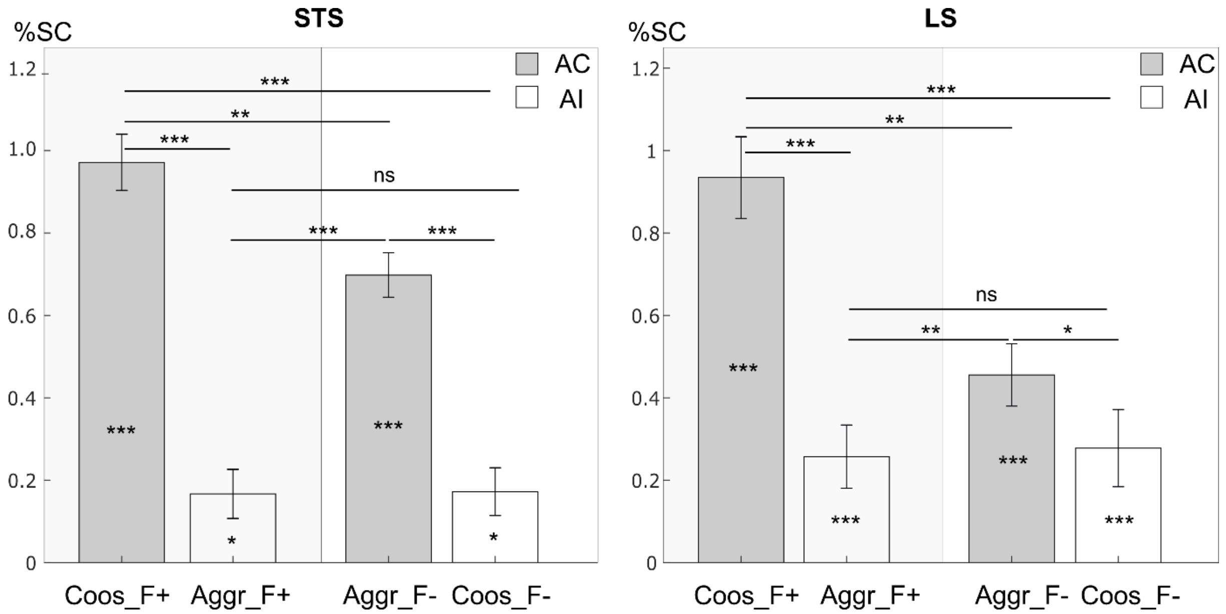

Supplementary Figure 6. Percentage of signal change (%SC) across all superior temporal sulcus and the lateral sulcus ROIs of both hemispheres (median + se, see ROI definition in figure 6, n= 14 ROIs for STS and n= 10 ROIs for LS), comparing auditory congruent and incongruent conditions vs fixation for the face context F+ and F- separately, and coos and aggressive calls independently. Note that auditory congruent (AC) stimuli for the positive face context (F+) are coos and incongruent auditory stimuli (AI) are aggressive calls (Aggr). For the negative face context (F-), this is reversed as coos are incongruent with respect to the context and aggressive calls are congruent. The specific types of coos and aggressive calls used in both F+ and F- contexts are identical. Statistical differences relative to fixation or across conditions in the F+ and F- face contexts are indicated as follows: ***, $p<0.001$; **, $p<0.01$; *, $p<0.05$; n.s., $p>0.05$ (Wilcoxon two-sided non-parametric test: STS: F+: Coos: n=280, Z=12.5, p<0.001= 6.8e-36; Aggr: n=280, Z=3.35, p=0.019; Coos vs Aggr: Z=7.67, p=1.7305e-14.<0.001 F-: Aggr: n=280, Z=10.01, p= 1.4e-23<0.001; Coos: n= 280, Z=3.34, p=0.019; Coos vs Aggr: Z=5.19, p=2.1e-07<0.001. Mann-Whitney two-sided non-parametric test: Coos_F+ vs Coos_F-: Z=7.63, p=2.2e-14<0.001; Aggr_F+ vs Aggr_F-: Z=5.29, p= 1.2e-07<0.001; Coos_F+ vs Aggr_F-: Z=3.13, p=0.002; Coos_F- vs Aggr_F+: Z=0.1, p=0.92. Wilcoxon two-sided non-parametric test: LS: F+: Coos: n=200, Z=8.69, p=3.6e-18<0.001; Aggr: n=200, Z=3.69, p=2.2e-04<0.001; Coos vs Aggr: Z=4.98, p=6.1e-07<0.001; F-: Aggr: n=200, Z=7.77, p= 8.1e-15<0.001; Coos: n=200, Z=4.43, p= 9.1e-06<0.001; Coos vs Aggr: Z=2.86, p=0.016; Mann-Whitney two-sided non-parametric test: Coos_F+ vs Coos_F-: Z=4.45, p= 8.5e-06<0.001; Aggr_F+ vs Aggr F-: Z=2.64, p=0.008; Coos_F+ vs Aggr_F-: Z=2.65, p=0.007; Coos_F- vs Aggr_F+: Z=0.65, p=0.51). This figure quantifies the effect strengths of the t-score maps presented in Figures 2 and 3.

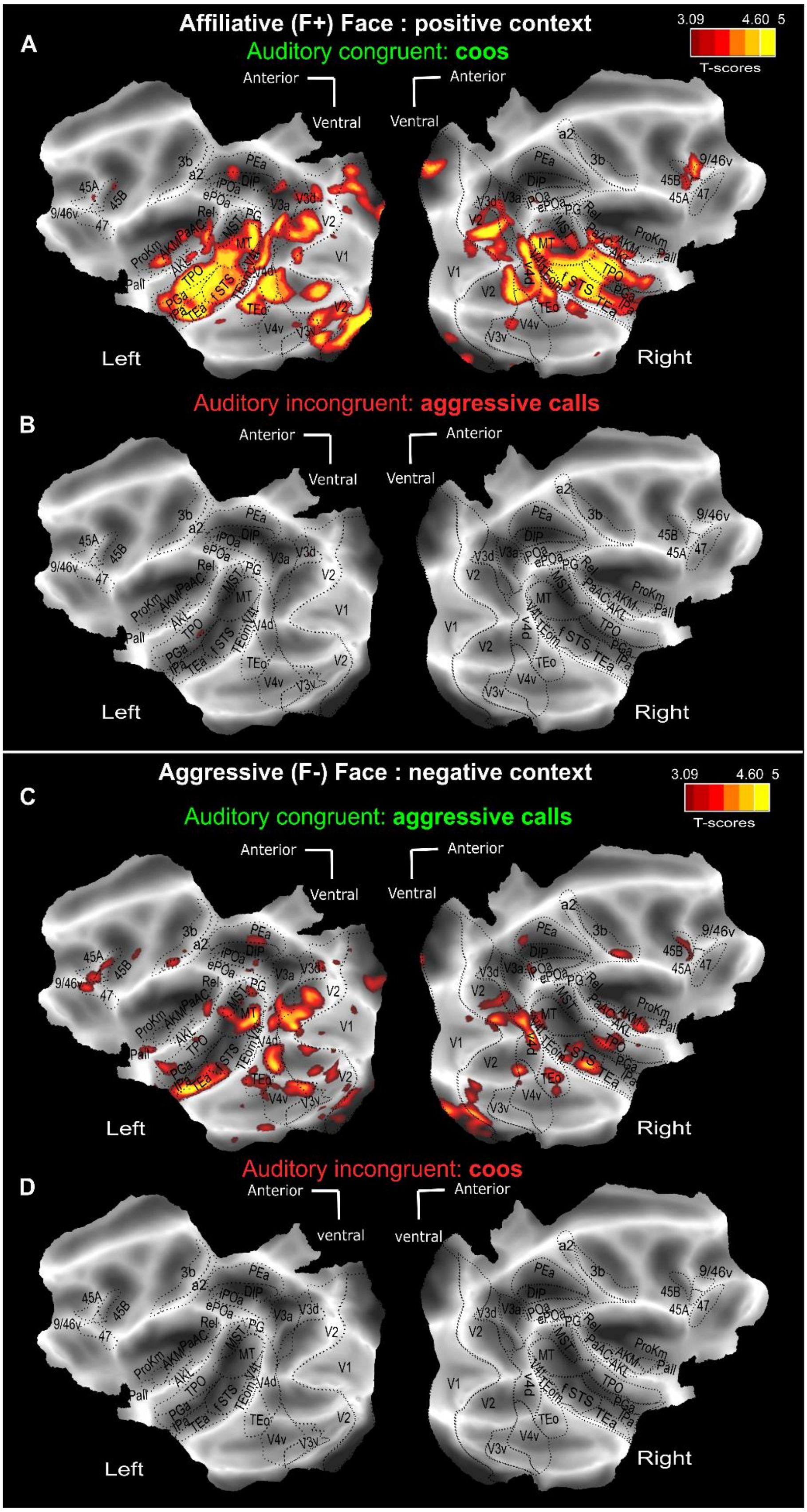

Supplementary Figure 7. All as in Figure 3, with the CIVM atlas (https://scalablebrainatlas.incf.org/macaque/CBCetal15, Calabrese et al., 2015)overlaid onto the cortical activations. PSC (median +/-s.e.) in each anatomical ROI described in Supplementary Table 2.

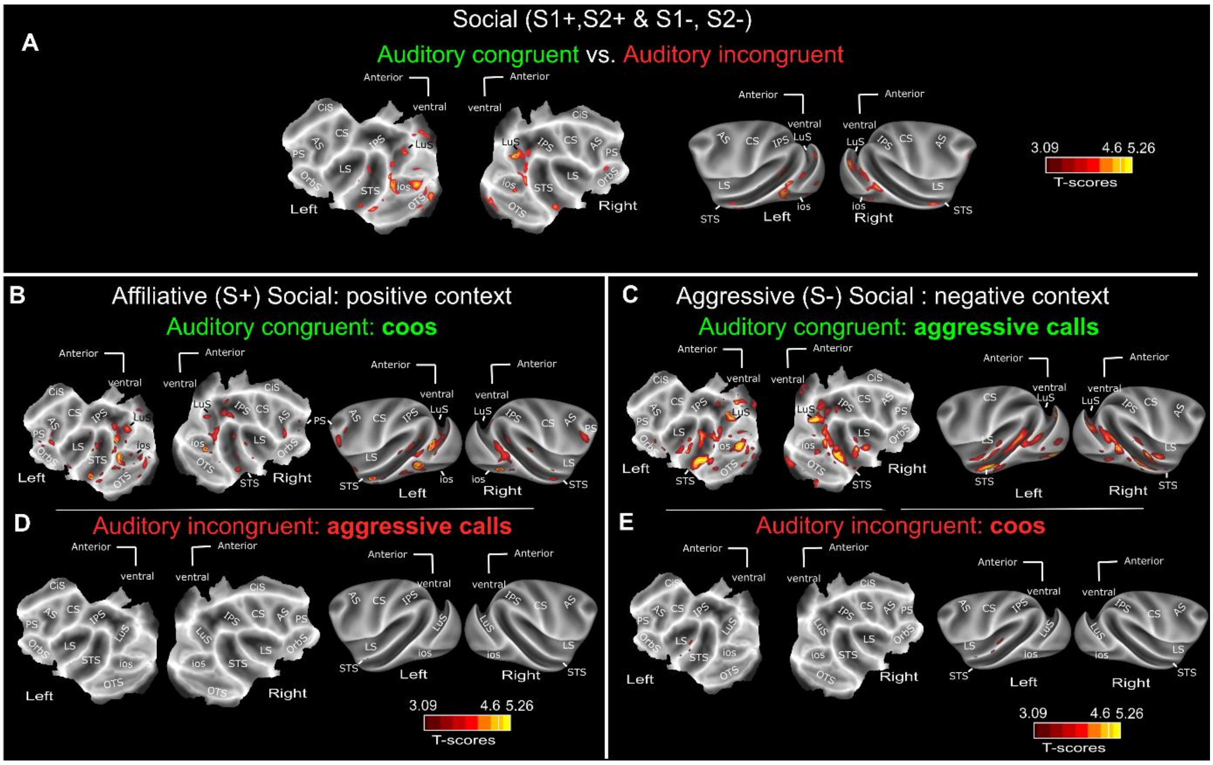

Supplementary Figure 8: Auditory activations depend on semantic congruence with social contexts. A) Whole-brain activation maps of the S1+, S2+ (social affiliative 1 & 2), S1- (social aggressive) and S2- (social escape) runs, for the *auditory congruent vs auditory incongruent* (relative to the visual context) contrast. B) Whole-brain activation map for the S+ (social affiliative, S1+&S2+) auditory congruent (coos, dark green, AC vs. Fx) and auditory incongruent (aggressive calls and screams, dark red, AI vs. Fx) conditions. C) Whole-brain activation map for the S- (social negative, S1-&S2-) auditory congruent (aggressive calls and screams, dark green, AC vs. Fx) and auditory incongruent (coos, dark red, AI vs. Fx) conditions. Darker shades of red indicate level of significance at p<0.001 uncorrected, t-score 3.09. Lighter shades of yellow and brown outlines indicate level of significance at p<0.05 FWE corrected, t-score 4.6. DF = [1, 10344] for Social, DF= [1, 5237] for S+ and DF= [1, 5107] for S-.

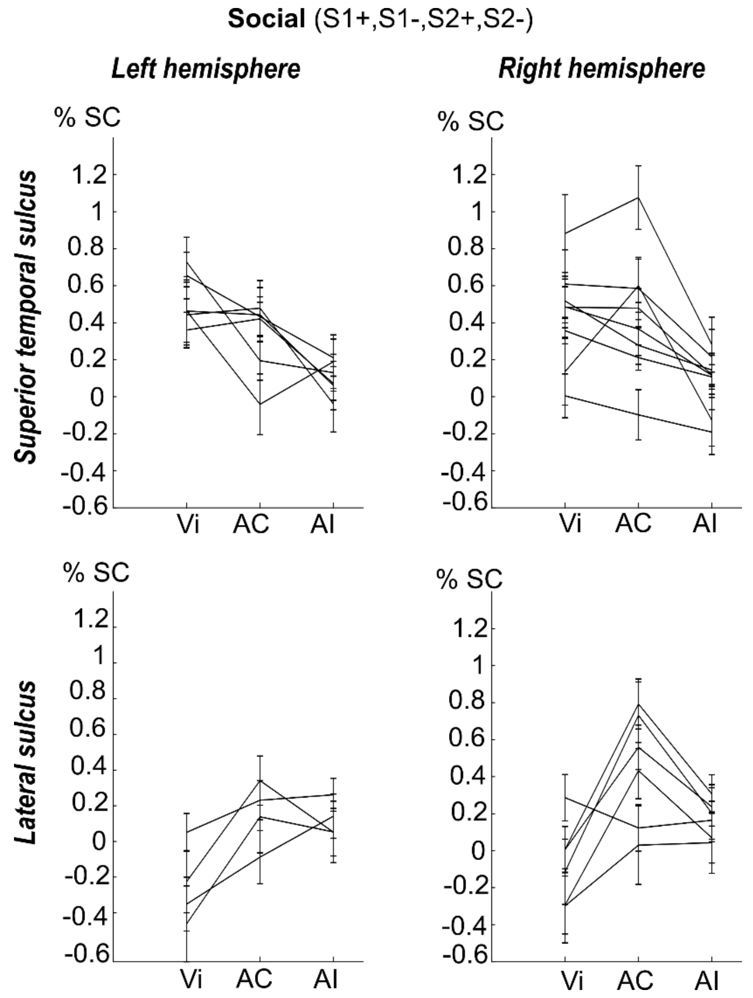

Supplementary Figure 9: Percentage of signal change (%SC) for selected left and right hemisphere ROIs in the lateral sulcus and in the superior temporal sulci. ROIs are 1.5mm spheres located at local peak activations of these two sulci. ROI location in the each of the left and right STS and LS is described in the bottom flat maps of figure 6. %SC (median +/- se) are presented for each ROI (8 in right STS, 6 in left STS, 4 in left and 6 in right lateral sulcus) for the social context for the contrast Visual vs fixation (Vi), Auditory congruent vs fixation (AC) and auditory incongruent vs fixation (AI).

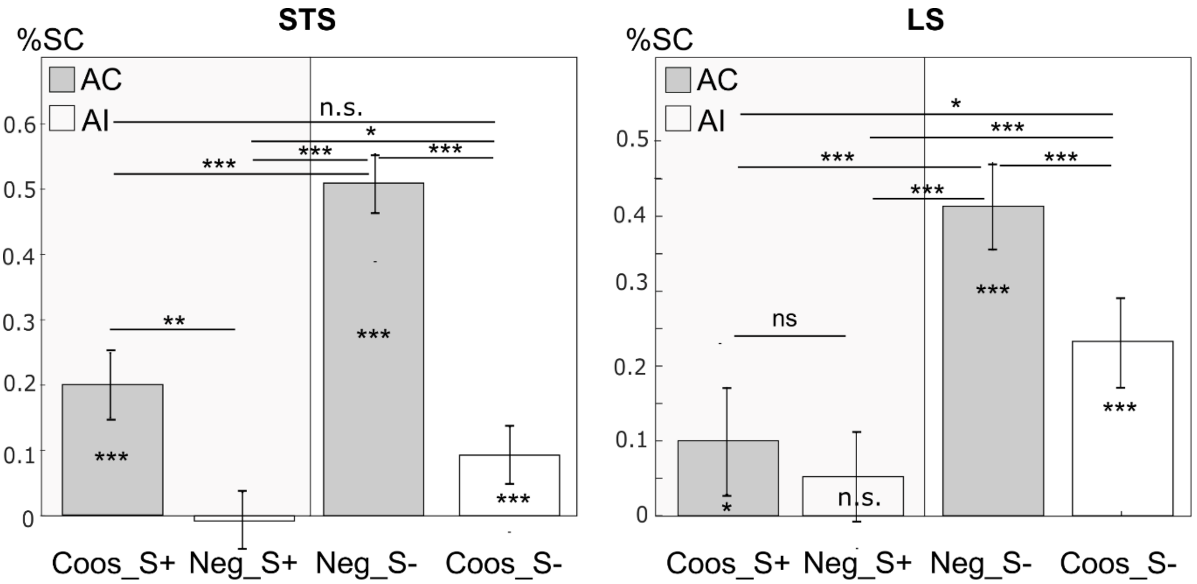

Supplementary Figure 10. Percentage of signal change (%SC) across all superior temporal sulcus and the lateral sulcus ROIs of both hemispheres (median + se, see ROI definition in figure 6, n= 14 ROIs for STS and n= 10 ROIs for LS), comparing auditory congruent and incongruent conditions vs fixation for the social context S+ and S- separately, and coos and negative calls (aggressive calls and screams) independently. Note that auditory congruent (AC) stimuli for the positive social context (S+) are coos and incongruent auditory stimuli (AI) are aggressive calls and screams (Neg). For the negative social context (S-), this is reversed as coos are incongruent with respect to the context and aggressive calls and screams are congruent. The specific types of coos and aggressive calls and screams used in both S+ and S- contexts are identical. Statistical differences relative to fixation or across conditions in the S+ and S- face contexts are indicated as follows: ***, p<0.001; **, p<0.01; *, p<0.05; n.s., p>0.05 (Wilcoxon two-sided non-parametric test: STS: S+: Coo: n=560, Z=5.30, p= 1.1e-07 ; Neg: n=560, Z=0.33, p=0.74; Coos vs Neg: Z=3.08, p=0.002. S-: Neg: n=546, Z=12.76, p=2.42e-37; Coo: n=546, Z=3.58, p=3.38e-04; Coos vs Neg: Z=5.72, p= 1.04e-08. Mann-Whitney two-sided non-parametric test: Coos_S+ vs Coos_S-: Z=0.99, p=0.31; Neg_S+ vs Neg_S-: Z=7.57, p= 3.5e-14; Coos_S+ vs Neg_S-: Z=4.23, p=2.24e-05; Coos_S- vs Neg_S+: Z=0.24, p=0.02. Wilcoxon two-sided non-parametric test: LS: S+: Coo: n=400, Z=2.04, p=0.03; Neg: n=400, Z=0.26, p=0.79; Coos vs Neg: Z=0.65, p=0.5. S-: Neg: n=390, Z=6.89, p= 5.6e-12; Coo: n=390, Z=6.62, p=3.4603e-11; Coos vs Neg: n=390, Z=3.86, p=2.3e-05; Mann-Whitney two-sided non-parametric test: Coos_S+ vs Coos_S-: Z=2.82, p=0.014; Neg_S+ vs Neg_S-: Z=4.24, p=2.2e-05; Coos_S+ vs Neg_S-: Z=3.01, p=4e-04; Coos_S- vs Neg_S+: Z=3.71, p=1.9e-04). This figure quantifies the effect strengths of the t-score maps presented in Figure 4.

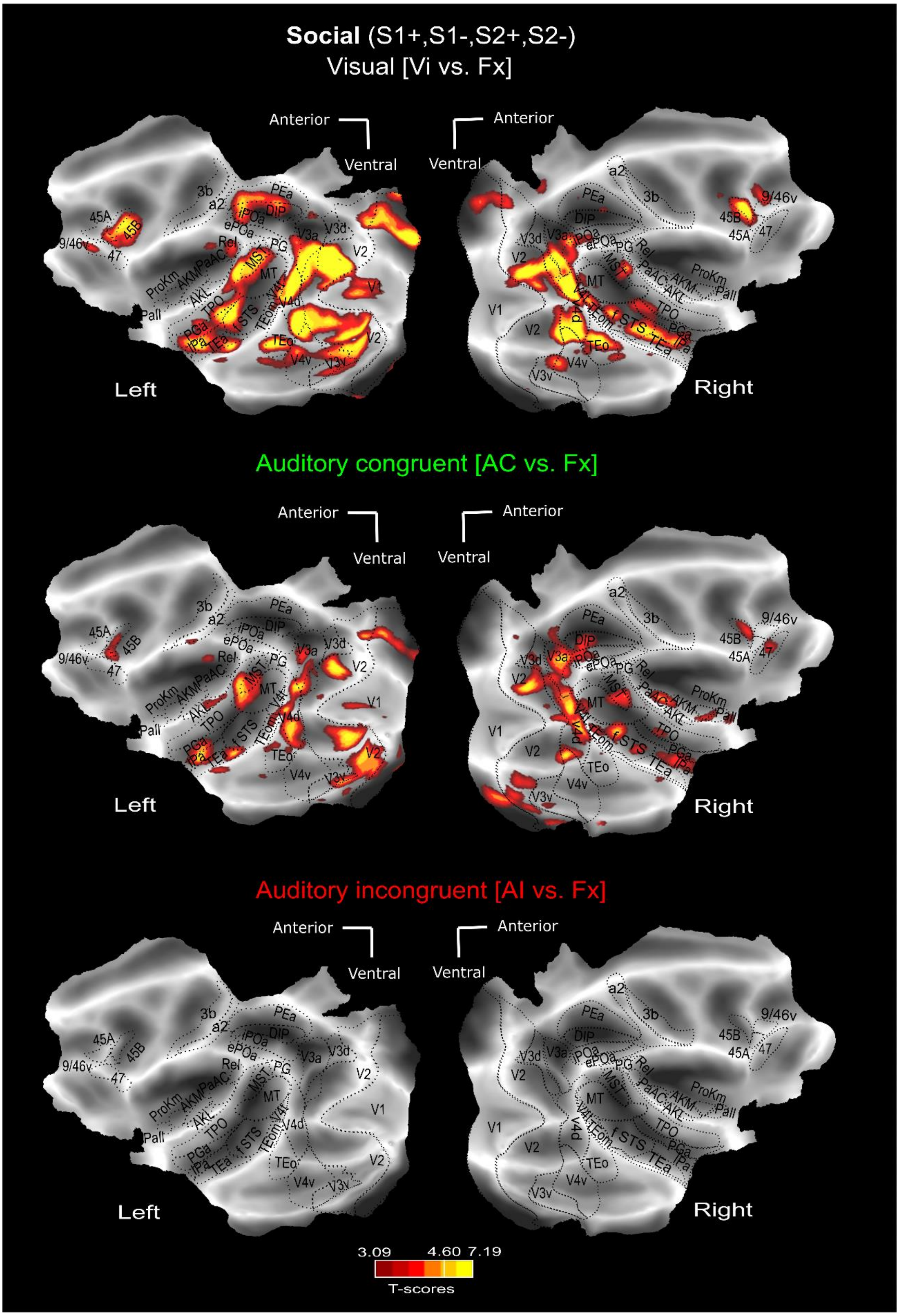

Supplementary Figure 11. All as in Figure 4, with the CIVM atlas (https://scalablebrainatlas.incf.org/macaque/CBCetal15, Calabrese et al., 2015) overlaid onto the cortical activations. PSC (median +/-s.e.) in each anatomical ROI described in Supplementary Table 3.

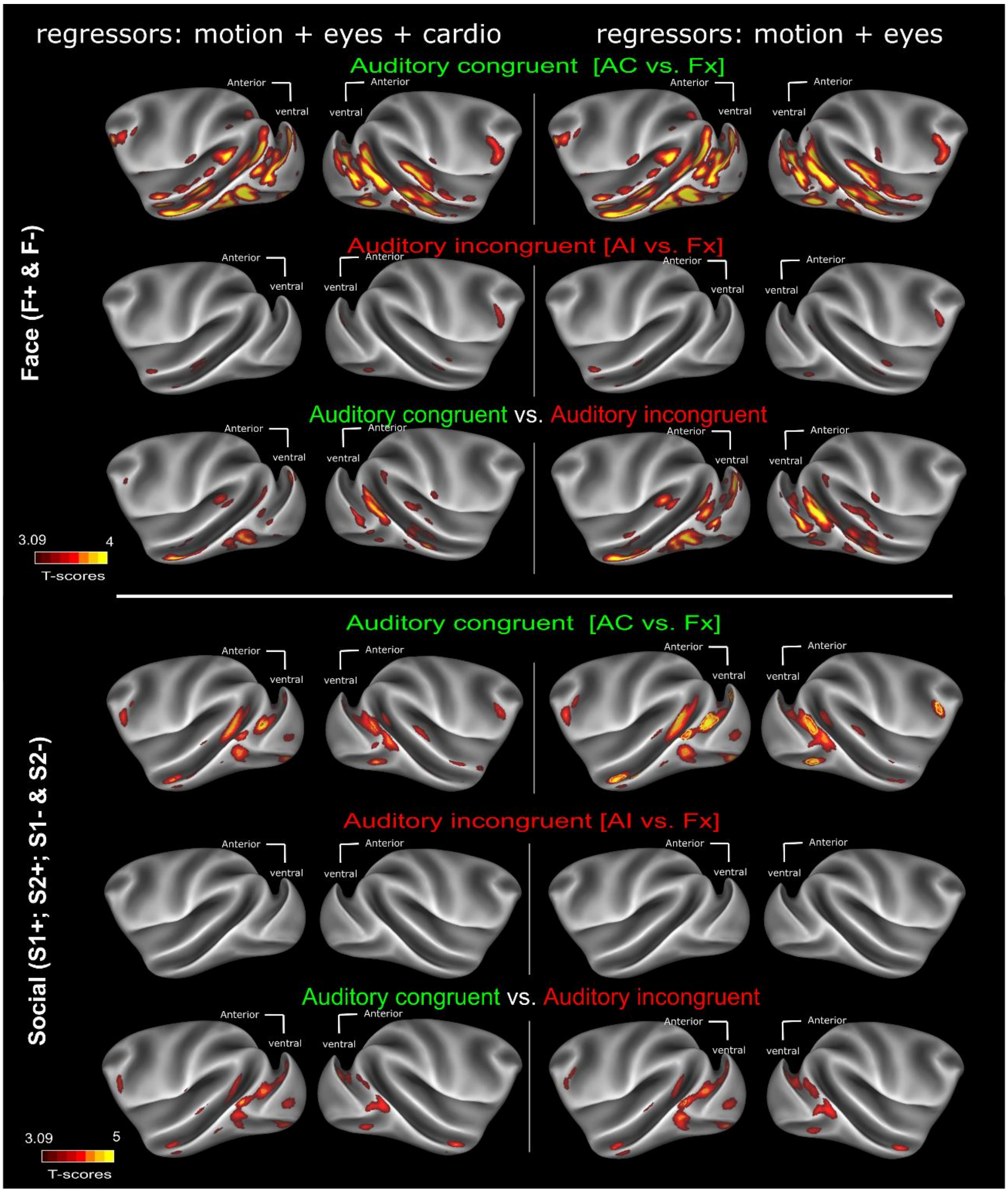

Supplementary Figure 12. Main contrast analyses of figures 2, 3 and 4 reproduced here including (left) or not (right) the heart rate as a regressor of non interest, for the Face (A) and Social (B) runs. Please note that because alignment triggers between heart rate signals and MRI scans are missing for a few sessions, this Supplementary analysis is run on fewer runs than those presented in the main paper (Face: 31 runs instead of 40; Social: 67 runs instead of 79). Maps are thresholded at $p<0.001$ uncorrected, t-score>3.09. DF= [1, 4015] for face and DF= [1, 8706] for social.

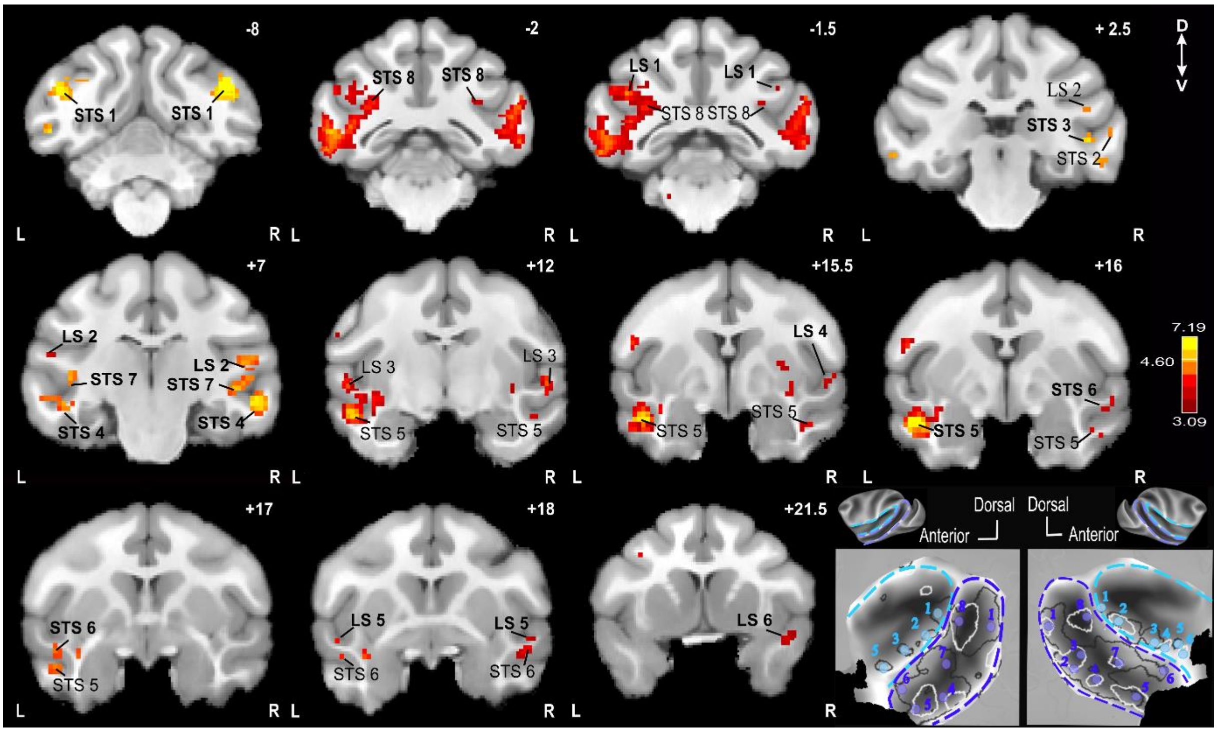

Supplementary Figure 13: **Peak activations that were used to define the ROIs of interest, on coronal slices, based on the AC vs. Fx contrast presented in (Figure 2 and Figure 4).** Antero-posterior level relative to the intra-aural line indicated at the top right corner of each slice, in millimeters. Bold fonts refer to the location peak. Normal fonts refer to cortical activation extending beyond the peak. Activation thresholds were varied on some sections in order to clearly show the existence of local activation maxima. Activation color-scale was however kept constant across all slices. Down-left panel: ROIs locations on flatmaps (lateral sulcus; light blue; superior temporal sulcus: dark blue), same conventions as in Figure 5.

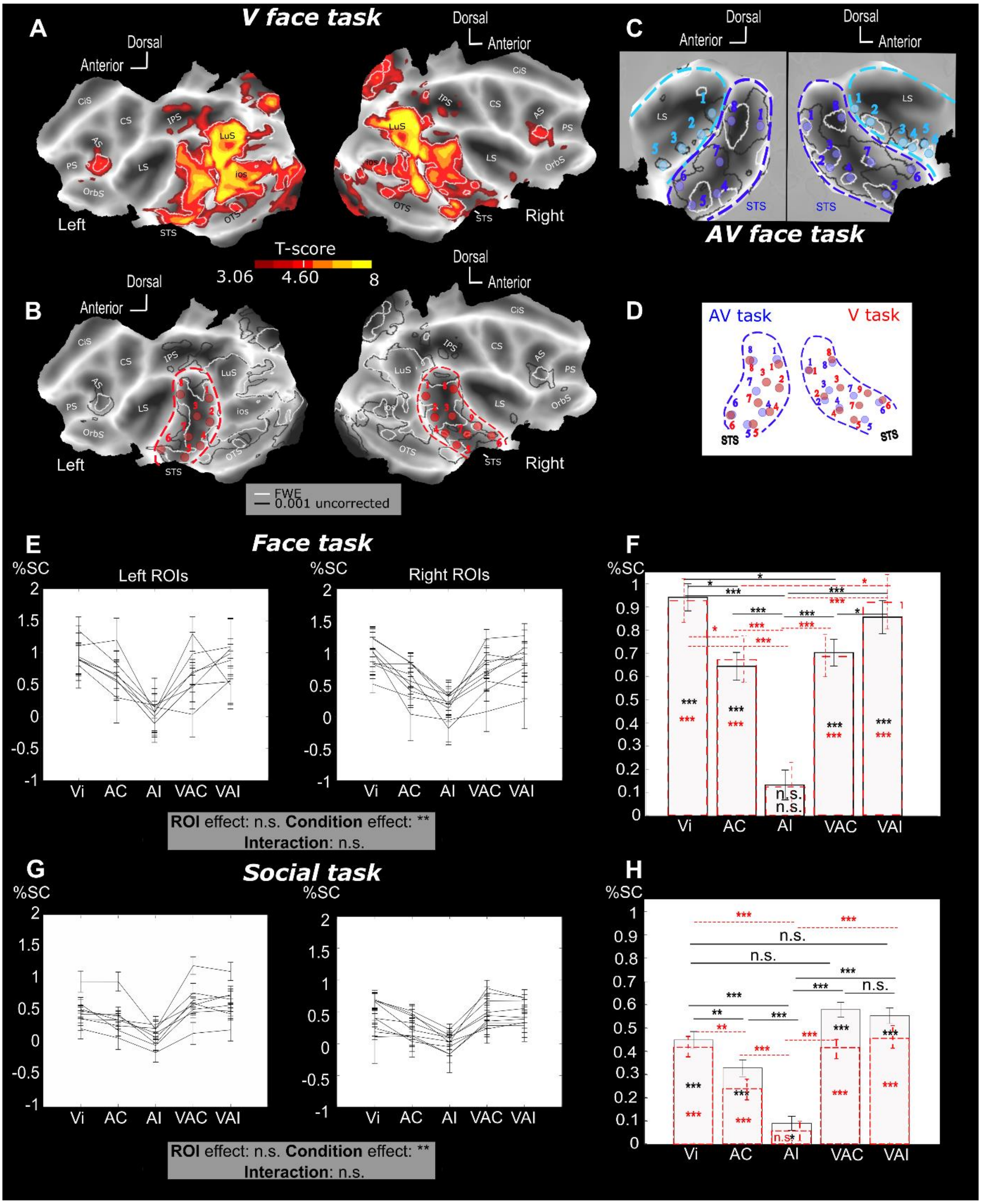

Supplementary Figure 14. (A) Whole-brain activation maps of a pure visual task cumulated over both monkeys, for lipsmack + aggressive blocks versus fixation contrast. Darker shades of red indicate level of significance at p<0.001 uncorrected, t-score>3.09. Lighter shades of yellow indicate level of significance at p<0.05 FEW corrected, t-score>4.6. DF= [1, 3121]. (B) ROIs are 1.5mm red spheres located at local peak activations of the pure visual task, in the STS. Left and right hemisphere numbering associate mirror ROIs. (C) ROIs are 1.5mm blue spheres located at local peak activations of the audio-visual task described in the main text. Left and right hemisphere numbering associate mirror ROIs,

reproducing main Figure 6. (D) STS location of the ROIs from the pure visual task (red) overlaid on those identified from the audio-visual task (blue). (E) Percentage signal change (%SC, median + se) in the audio-visual face context and each condition of interest vs fixation (V: visual, AC: auditory congruent, AI: auditory incongruent, VAC: visuo-auditory congruent, VAI: visuo-auditory incongruent) are presented for each ROI defined from the pure visual task. No ROI effect $p>0.05$, condition effect **, $p<0.01$, no interaction effect $p>0.05$ (Friedman two-sided non-parametric test). This reproduces the results presented in main Figure 6 (n= 14 ROIs for STS and n= 10 ROIs for LS), with ROIs defined in the visual task. (F) Percentage of signal change (%SC) across all ROIs (extracted from the pure visual task) of superior temporal sulcus in both hemispheres (median + se, n= 14 ROIs for STS and n= 10 ROIs for LS), comparing the different conditions of interest vs fixation in the audio-visual face context (V: visual, AC: auditory congruent, AI: auditory incongruent, VAC: visuo-auditory congruent, VAI: visuo-auditory incongruent). Black: all ROIs; red: only ROIs that do not overlap with the ROIs defined from the main audio-visual task. Statistical differences relative to fixation or between conditions in the face context and indicated as follows: ***, $p<0.001$; **, $p<0.01$; *, $p<0.05$, n.s., $p>0.05$ (Wilcoxon two-sided non-parametric test). (G) Same as E, but for the audio-visual social task. This reproduces the results presented in main Figure 6, with ROIs defined in an independent visual task. (H) Same as F, but for the audio-visual social task.

Supplementary note associated with Supplementary Figure 14. We use a visual task consisting of runs composed of pseudorandom alternations of blocks of faces with different facial expressions (aggressive, neutral, lip-smacking, scared) and fixation. We use this task to define ROIs that are independent from the audio-visual tasks presented in the main paper and we reproduce our main observation that in these new ROIs, the %SC in the AI condition (relative to fixation), is significantly lower than in all the other conditions. This thus allows to generalize our observations at locations independent from the peak activations identified in the main audio-visual task. Specifically, we use the aggressive+lipsmack vs. fixation contrast, thus using the same visual stimuli as in the audio-visual face task, to identify face responsive activations (Supplementary Figure 15a). We identified the activation local maxima observed in the STS with this contrast (Supplementary Figure 15b). Figure S15d shows the location of these peaks (red) relative to those defined in the audio-visual task (blue, Supplementary Figure 15c). While some of these ROIs closely overlap with those identified in the audio-visual task, others don't, suggesting a specialization for social audio-visual processing in these latter ROIs. In both the audio-visual face (Supplementary Figure 15e, Supplementary Figure 15f) and social task (Supplementary Figure 15g, Supplementary Figure 15h), AI %SC relative to fixation is, over all ROIs, significantly lower than %SC in all other conditions, including when only considering the ROIs from the visual task that are non-overlapping with the ROIs from the audio-visual task (Supplementary Figure 15g, Supplementary Figure 15h, red). Overall, this thus fully rules out any possible concern about double dipping and indicates that the results reported in the main manuscript are not idiosyncratic to the ROI definition.

Design of the pure Visual runs

The design of the visual runs was similar to that of the audio-visual run design, organized in blocks, except for the fact that all blocks were pure visual blocks and varied as a function of facial emotions. The six possible 16 s blocks were: fixation (Fx), lipsmack, scared monkey faces, aggressive monkey faces, neutral monkey faces and scrambled monkey faces. As for the audio-visual runs, each block consisted in an alternation of 500 ms stimuli (except for lip smacks, 1s dynamic stimuli succession) of the same emotional category, as was the case in the main task.

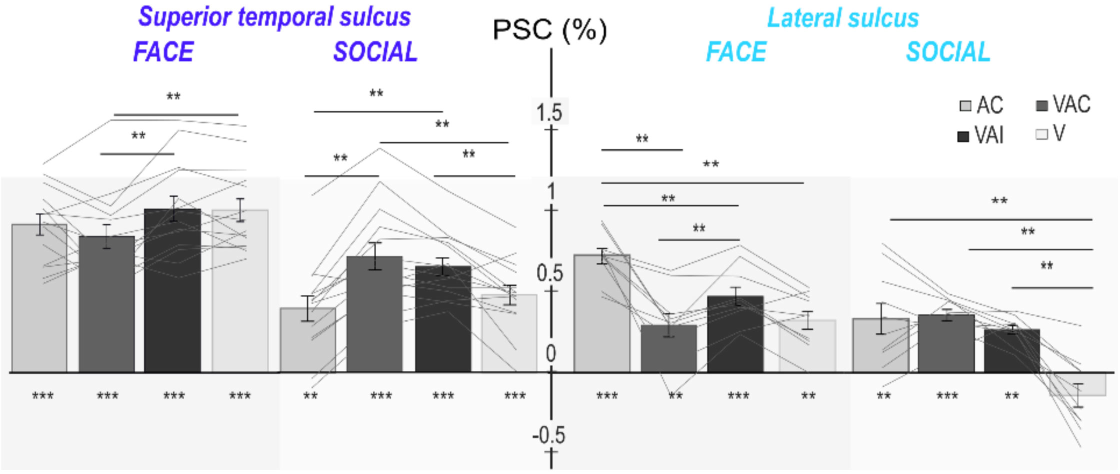

Supplementary Figure 15: **Percentage of signal change (%SC) across all lateral sulcus (light blue) and superior temporal sulci (dark blue) ROIs of both hemispheres, comparing unimodal and multimodal congruent and incongruent conditions** (median ± s.e., n= 14 ROIs for STS and n= 10 ROIs for LS). Statistical differences relative to fixation are between conditions are indicates as follows: ***, p<0.001, **, p<0.01, *, p<0.05 (Wilcoxon two-sided non-parametric test, STS: FACE: AC: Z= 16.14, p<0.001; V: Z= 19.35, p<0.001; VAC : Z=19.13, p<0.001; VAI : Z=17.25, p<0.001; AC vs V: Z=1.68, p=0.09 ; V vs VAC: Z=1.88, p<0.01 ; VAC vs VAI: Z=1.86, p<0.01. SOCIAL: AC: Z= 11.49, p<0.01; V: Z= 14.87, p<0.001; VAC: Z=18.49, p<0.001; VAI: Z=15.98, p<0.001; AC vs VAC: Z=5.35, p<0.01; AC vs VAI: Z=4.06, p<0.01; V vs VAC: Z=2.64, p<0.01; V vs VAI: Z=2.48, p<0.01. LS: FACE: AC: Z= 11.65, p<0.001; V: Z= 4.84, p<0.01; VAC: Z=3.92, p<0.001; VAI: Z=6.28, p<0.001; AC vs V: Z=3.97, p<0.01; AC vs VAC: Z=5.31, p<0.01; AC vs VAI: Z=5.31, p<0.01, VAC vs VAI: Z= 2.05 p<0.01. SOCIAL: AC: Z= 5.86, p<0.01; V: Z= -0.7, p=0.45; VAC: Z=8.19, p<0.001; VAI: Z=7.26, p<0.001; AC vs V: Z=4.7, p<0.01; VAC vs V: Z= 5.47 p<0.01; VAI vs V: Z=4.66 p<0.01).

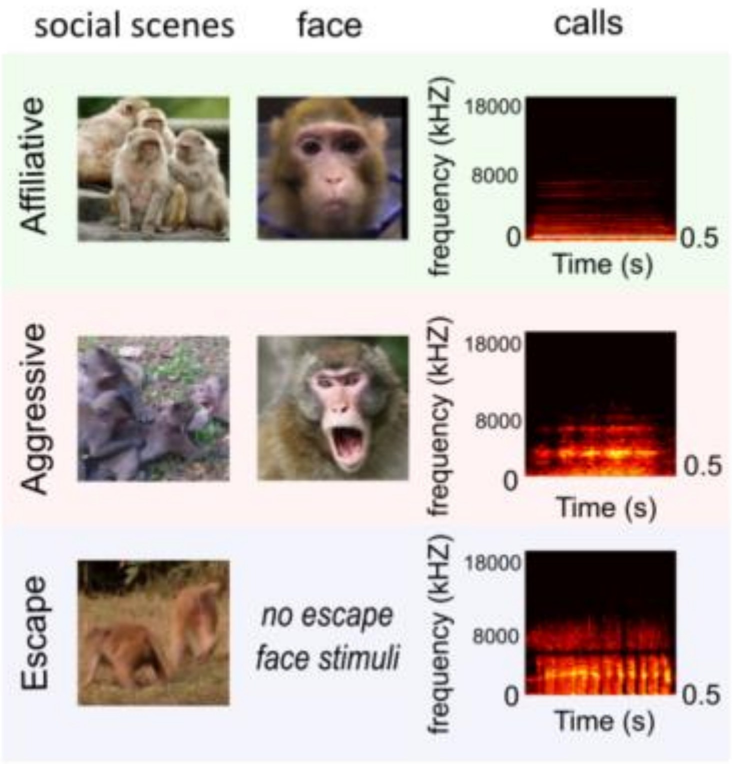

Supplementary Figure 16: Example of visual and auditory stimuli. At the right are shown examples of visual stimuli used for social and face contexts. On the left, congruent calls spectrograms are associated to the visual stimuli are shown. The affiliative call is a coo, the aggressive congruent auditory stimulus is an aggressive call and the escape call is a scream. Stimuli were not strictly normalized in terms of in low visual and auditory feature properties, thus making their social meaning the dominant cue across the different stimuli of a given category. Stimuli were extracted from videos collected by the Ben Hamed lab, as well as by Marc Hauser on Cayo Santiago, Puerto Rico.

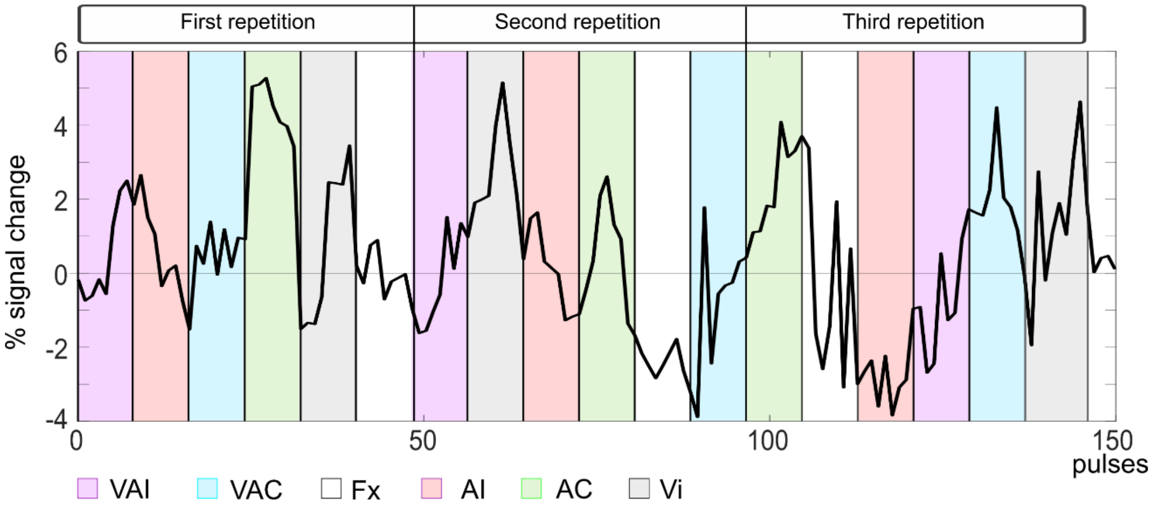

Supplementary Figure 17. Median time courses in percent signal change extracted from the STS ROIs in a representative Face run. Time courses were averaged across monkeys, hemispheres and ROIs for runs having the same bloc organization. Block conditions (16s) are indicated by different colors: VAI in purple, VAC in cyan, fixation in white, AI in red, AC in green and Vi in gray. Specifically, time courses were extracted thanks to MarsBaR (Brett et al., 2002), for each STS ROIs and for each of the selected Face context runs. We averaged the signal across these ROIs and across runs with the same block sequence. This includes 4 runs (one per F+ and F- face context and per monkey). We converted the signal in percent signal change ((signal-signal's median / signal's median) x 100). We then inverted the signal to account for the fact that the MION produces a decrease in signal following neuronal activation.

Supplementary References

1. Calabrese, E. *et al.* A diffusion tensor MRI atlas of the postmortem rhesus macaque brain. *NeuroImage* **117**, 408–416 (2015).
2. Brett, M., Anton, J.-L., Valabregue, R. & Poline, J.-B. Region of interest analysis using an SPM toolbox. 1.
