# Supplementary material for: Socially meaningful visual context either enhances or inhibits vocalisation processing in the macaque brain
Source: Nat Commun. 2022 Aug 19;13:4886. doi: 10.1038/s41467-022-32512-9 (PMC9391382; doi:10.1038/s41467-022-32512-9)
Supplement: Supplementary file 3 — Reporting Summary [file 41467_2022_32512_MOESM3_ESM.pdf]

Corresponding author(s): Ben Hamed Suliann

Last updated by author(s): 13/07/2022

# Reporting Summary

Nature Portfolio wishes to improve the reproducibility of the work that we publish. This form provides structure for consistency and transparency in reporting. For further information on Nature Portfolio policies, see our Editorial Policies and the Editorial Policy Checklist.

## Statistics

For all statistical analyses, confirm that the following items are present in the figure legend, table legend, main text, or Methods section.

| n/a | Confirmed | |
|---|---|---|
| ☐ | ☒ | The exact sample size ($n$) for each experimental group/condition, given as a discrete number and unit of measurement |
| ☐ | ☒ | A statement on whether measurements were taken from distinct samples or whether the same sample was measured repeatedly |
| ☐ | ☒ | The statistical test(s) used AND whether they are one- or two-sided<br>*Only common tests should be described solely by name; describe more complex techniques in the Methods section.* |
| ☐ | ☒ | A description of all covariates tested |
| ☐ | ☒ | A description of any assumptions or corrections, such as tests of normality and adjustment for multiple comparisons |
| ☐ | ☒ | A full description of the statistical parameters including central tendency (e.g. means) or other basic estimates (e.g. regression coefficient) AND variation (e.g. standard deviation) or associated estimates of uncertainty (e.g. confidence intervals) |
| ☐ | ☒ | For null hypothesis testing, the test statistic (e.g. $F$, $t$, $r$) with confidence intervals, effect sizes, degrees of freedom and $P$ value noted<br>*Give P values as exact values whenever suitable.* |
| ☒ | ☐ | For Bayesian analysis, information on the choice of priors and Markov chain Monte Carlo settings |
| ☒ | ☐ | For hierarchical and complex designs, identification of the appropriate level for tests and full reporting of outcomes |
| ☐ | ☒ | Estimates of effect sizes (e.g. Cohen's $d$, Pearson's $r$), indicating how they were calculated |

*Our web collection on statistics for biologists contains articles on many of the points above.*

## Software and code

Policy information about availability of computer code

| | |
|---|---|
| Data collection | Eye position (X, Y, right eye) was recorded thanks to a pupil-corneal reflection video-tracking system (EyeLink at 1000 Hz, SR-Research) interfaced with a program for stimulus delivery and experimental control (EventIDE®). The MRI scans were performed on a 3T Magnetom Prisma system (Siemens Healthineers, Erlangen, Germany). |
| Data analysis | fMRI data were pre-processed and analysed using AFNI (Cox, 1996), FSL (Jenkinson et al., 2012; Smith et al., 2013), SPM software (version SPM12, Wellcome Department of Cognitive Neurology, London, UK, https://www.fil.ion.ucl.ac.uk/spm/software/), JIP analysis toolkit (http://www.nitrc.org/projects/jip), Workbench (https://www.humanconnectome.org/software/get-connectome-workbench, V1.5.0) and Marsbar SPM toolbox (marsbar.sourceforge.net, release 0.42). Heart-rate was extracted from videos based on the published method in (Froesel et al., 2020). |

For manuscripts utilizing custom algorithms or software that are central to the research but not yet described in published literature, software must be made available to editors and reviewers. We strongly encourage code deposition in a community repository (e.g. GitHub). See the Nature Portfolio guidelines for submitting code & software for further information.

## Data

Policy information about availability of data

All manuscripts must include a data availability statement. This statement should provide the following information, where applicable:

- Accession codes, unique identifiers, or web links for publicly available datasets
- A description of any restrictions on data availability
- For clinical datasets or third party data, please ensure that the statement adheres to our policy

The data that support the findings of this study are available from the corresponding author upon reasonable request. Data are still being analysed for other

purposes and cannot be made publically available at this time.

The code that supports the findings of this study is available from the corresponding author upon reasonable request. The code is still being used for other purposes and cannot be made publically available at this time.

# Field-specific reporting

Please select the one below that is the best fit for your research. If you are not sure, read the appropriate sections before making your selection.

- [x] Life sciences
- [ ] Behavioural & social sciences
- [ ] Ecological, evolutionary & environmental sciences

For a reference copy of the document with all sections, see nature.com/documents/nr-reporting-summary-flat.pdf

# Life sciences study design

All studies must disclose on these points even when the disclosure is negative.

| | |
|---|---|
| Sample size | In non-human primate experiments, sample sizes in single group studies are typically set to two since they are limited by both ethical and practical constraints (Bliss-Moreau et al, 2021). In order to meet the ethical requierements of reduction, following the recommandation of the ethical committee, we describe our findings and perform our statistical tests in two macaque monkeys but 10 repeats of data collection per condition per monkey. Significance was assessed both at the group and at the individual level. |
| Data exclusions | We excluded runs based on the monkey's fixation quality during each run. 85% within the eye fixation tolerance window were required. |
| Replication | The observations made in this study are highly robust as they are reproduced over six sets of independent behavioral contexts, involving distinct associations of visual and auditory social information, in both animals and in both MRI data and cardiac data. |
| Randomization | The different contexts of tasks were presented randomly during the MRI sessions. In the task design a pseudo-randomization was implemented such that each condition block in each repetition was presented in a randomized order. Thus monkeys could not anticipate the sequence of stimuli. The pseudo-randomization was also implemented such that, across all repetitions and all runs for a given context, each condition block was, on average, preceded by the same number of blocks from the other conditions. |
| Blinding | There was no specific group allocation and no biais could come from the investigators due to the fact that the monkeys were performing a passive task. Thus, this study did not require blinding. |

# Reporting for specific materials, systems and methods

We require information from authors about some types of materials, experimental systems and methods used in many studies. Here, indicate whether each material, system or method listed is relevant to your study. If you are not sure if a list item applies to your research, read the appropriate section before selecting a response.

## Materials & experimental systems

| n/a | Involved in the study | |
|---|---|---|
| x | | Antibodies |
| x | | Eukaryotic cell lines |
| x | | Palaeontology and archaeology |
| | x | Animals and other organisms |
| x | | Human research participants |
| x | | Clinical data |
| x | | Dual use research of concern |

## Methods

| n/a | Involved in the study | |
|---|---|---|
| x | | ChIP-seq |
| x | | Flow cytometry |
| | x | MRI-based neuroimaging |

## Animals and other organisms

Policy information about studies involving animals; ARRIVE guidelines recommended for reporting animal research

| | |
|---|---|
| Laboratory animals | Two male rhesus monkeys (Macaca mulatta) participated in the study (T, 15 years, 10kg and S, 12 years, 11kg). |
| Wild animals | The study did not involved wild animals. |
| Field-collected samples | The study did not involve samples collected from the field. |
| Ethics oversight | The project was authorized by the French Ministry for Higher Education and Research (project no. 2016120910476056 and 1588-2015090114042892) in accordance with the French transposition texts of Directive 2010/63/UE. This authorization was based on ethical evaluation by the French Committee on the Ethics of Experiments in Animals (C2EA) CELYNE registered at the national level as C2EA number 42. |

Note that full information on the approval of the study protocol must also be provided in the manuscript.

# Magnetic resonance imaging

## Experimental design

| | |
|---|---|
| Design type | Block design |
| Design specifications | Each run started with 10 s of fixation in the absence of sensory stimulation followed by three repetitions of a pseudo-randomized sequence containing six 16 s blocks: fixation (Fx), visual (Vi), auditory congruent (AC), auditory incongruent (AI), congruent audio-visual (VAC) and incongruent audio-visual (VAI). The pseudo-randomization was implemented such that each block in each repetition was presented in a randomized order. Thus monkeys could not anticipate the sequence of stimuli. In addition, the initial blocks were either a visual block (Vi, VAC, VAI), or a fixation block followed by a visual block (Vi, VAC or VAI), such that context was set by visual information early on in each run. As a result, pure auditory blocks were always presented after a visual block and could thus be defined as congruent or incongruent to the visual information characterizing the block. Pseudo-randomization was also implemented such that, across all repetitions and all runs for a given context, each block was, on average, preceded by the same number of blocks from the other conditions. Quite crucially to the results presented in this work, in 66% of the times, both AI and AC conditions were preceded by blocks involving visual stimulation (Vi, VAC and VAI). Last, each block (except the fixation block) consisted in an alternation of 500 ms stimuli (except for lipsmacks, 1s dynamic stimuli succession) of the same semantic category (see Stimuli section below), in the visual, auditory or audio-visual modalities. In each block, 32 stimuli were presented randomly (16 for lipsmack). Each run ended by 10 s of fixation in the absence of any sensory stimulations. |
| Behavioral performance measures | This task was a passive task. The monkeys were requiered only to fixate a central cross on the screen. |

## Acquisition

| | |
|---|---|
| Imaging type(s) | functional, anatomical |
| Field strength | 3 tesla |
| Sequence & imaging parameters | T1-weighted anatomical images were acquired for each subject using a magnetization-prepared rapid gradient-echo (MPRAGE) pulse sequence. Spatial resolution was set to 0.5 mm, with TR= 3000 ms, TE=3.62 ms, Inversion Time (TI) =1100 ms, flip angle=8°, bandwidth=250 Hz/pixel, 144 slices. T2-weighted anatomical images were acquired per monkey, using a Sampling Perfection with Application optimized Contrasts using different flip angle Evolution (SPACE) pulse sequence. Spatial resolution was set to 0.5 mm, with TR= 3000 ms, TE= 366.0 ms , flip angle=120°, bandwidth=710 Hz/pixel, 144 slices.<br>Functional images were gradient-echoechoplanar images covering the whole brain (TR=2000 ms; TE=18 ms; 37 sagittal slices; resolution: 1.25x1.25x1.38 mm anisotropic voxels; flip angle=90°, bandwidth=1190 Hz/pixel) |
| Area of acquisition | Whole brain scans were used. |

Diffusion MRI ☐ Used ☒ Not used

## Preprocessing

| | |
|---|---|
| Preprocessing software | Data were pre-processed and analysed using AFNI (Cox, 1996), FSL (Jenkinson et al., 2012; Smith et al., 2013), SPM software (version SPM12, Wellcome Department of Cognitive Neurology, London, UK, https://www.fil.ion.ucl.ac.uk/spm/software/), JIP analysis toolkit (http://www.nitrc.org/projects/jip) and Workbench (https://www.humanconnectome.org/software/get-connectome-workbench). Functional volumes were corrected for head motion and slice time and skull-stripped. They were then linearly realigned on the T2-weighted anatomical image with flirt from FSL, the image distortions were corrected using nonlinear warping with JIP. A spatial smoothing was applied with a 3-mm FWHM Gaussian Kernel. |
| Normalization | T1-weighted and T2-weighted anatomical images were processed according to the HCP pipeline (Autio et al., 2020; Glasser et al., 2013) |
| Normalization template | Data were normalized into the MY19 Atlas (Donahue et al., 2016) |
| Noise and artifact removal | Head motion and eye movements were included as covariate of no interest in our general linear model analysis. |
| Volume censoring | We did not use volume censoring. |

## Statistical modeling & inference

| | |
|---|---|
| Model type and settings | We used the task conditions as fixed effects in our general linear model analysis (GLM). Head motion and eye movements were included as covariate of no interest in our GLM analysis. |
| Effect(s) tested | We tested the effet of visual, auditory and audio-visual stimulation on the brain in different emotional contexts. We compared these different conditions with a control block in which no visual or auditory stimulation were displayed. |

Specify type of analysis: ☐ Whole brain ☐ ROI-based ☒ Both

Anatomical location(s): ROIs were defined as 1.5 mm diameter spheres centred around the local peaks of activation.

Statistic type for inference (See Eklund et al. 2016): We used voxelwise inference.

Correction: Fixed effect individual analyses were performed for each monkey, with a level of significance set at $p<0.05$ corrected for multiple comparisons (FWE, t-scores 4.6) and $p<0.001$ (uncorrected level, t-scores 3.09).

## Models & analysis

| n/a | Involved in the study |
|---|---|
| ☒ | ☐ Functional and/or effective connectivity |
| ☒ | ☐ Graph analysis |
| ☒ | ☐ Multivariate modeling or predictive analysis |
